# Blends of Cyanate Ester and Phthalonitrile–Polyhedral Oligomeric Silsesquioxane Copolymers: Cure Behavior and Properties

**DOI:** 10.3390/polym11010054

**Published:** 2019-01-01

**Authors:** Xiaodan Li, Fei Zhou, Ting Zheng, Ziqiao Wang, Heng Zhou, Haoran Chen, Lin Xiao, Dongxing Zhang, Guanhui Wang

**Affiliations:** 1 School of Chemistry and Chemical Engineering, Jinggangshan University, No 28, Xueyuan Road, Qingyuan District, Ji’an 343009, China; lixiaodanlixiaodan@126.com; 2 School of Materials Science and Engineering, Harbin Institute of Technology, Harbin 150001, China; angel.flyfly@hotmail.com (F.Z.); zthappy1127@gmail.com (T.Z.); 3 Harbin FRP Institute, Harbin 150029, China; hit_wzq@126.com (Z.W.); chr0526@163.com (H.C.); 4 Institute of Chemistry, Chinese Academy of Sciences, No.2 Haidian District, Beijing 100190, China; zhouheng@iccas.ac.cn

**Keywords:** phthalonitrile polymers, phthalonitrile-polyhedral oligomeric silsesquioxane copolymers, cyanate ester, blends, thermal properties

## Abstract

Blends of cyanate ester and phthalonitrile–polyhedral oligomeric silsesquioxane copolymers were prepared, and their cure behavior and properties were compared via differential scanning calorimetry (DSC) analysis, thermogravimetric (TG) analysis, dynamic mechanical analysis, Fourier-transform far-infrared (FTIR) spectroscopy, and rheometric studies. The copolymer blends showed high chemical reactivity, low viscosity, and good thermal stability (TG temperatures were above 400 °C). The glass-transition temperature of the blends increased by at least 140 °C compared to cyanate ester resin. The blends are suitable for preparing carbon-fiber-reinforced composite materials via a winding process and a prepreg lay-up process with a molding technique. The FTIR data showed that the polymerization products contained triazine-ring structures that were responsible for the superior thermal properties.

## 1. Introduction

Phthalonitrile-based composites [1,2,3,4,5,6,7,8] with high temperature resistance, ablation resistance, low flammability, and high strength have great potential in the aerospace sector as components for maintaining airframe loads for the next generation of aeronautical and space vehicle systems. They are one of the few to meet the United States Navy’s stringent requirements under MIL-STD-2031 for the usage of polymer composites aboard Navy submarines. The model compound of the phthalonitrile monomer, 4,4′-bis(3,4-dicyanophenoxy)biphenyl (BPh), was first discovered at the U.S. Naval Research Laboratory [9,10,11,12,13,14,15,16]. However, the high melting temperature and poor solubility of the monomer limit its application. Many phthalonitrile monomers [17,18,19,20,21] have been synthesized to lower the melting temperature and improve their processing properties and use in resin-infusion fabrication.

We previously [22,23] introduced polyhedral oligomeric silsesquioxane (POSS) into a phthalonitrile system and prepared POSS–phthalonitrile copolymers. The POSS reagents consist of an inorganic silsequioxane cage and have multiple reactive groups that can react with the cyanate group at high temperatures. They provide a unique opportunity to prepare nanocomposites with truly molecular dispersion of the inorganic fillers. Thus, the POSS copolymers are easily prepared at low temperatures with a short curing time. Concurrently, their enhanced properties, such as higher *T*_g_ temperature, oxidative resistance, improved mechanical property, and fire resistance, have been shown in many thermoplastics, such as polyethylene, polypropylene, polycarbonate, as well as thermosetting polymers. These include polyimides, epoxy resins, polyurethane, and cyanate ester resins [24,25,26,27,28,29,30,31,32]. However, POSS–phthalonitrile copolymers are still applicable in resin-infusion fabrication.

To expand the use of resins in the field of composite materials, an urgent problem to overcome is the development of resin systems suitable for the winding process and prepreg lay-up process. The key to solving this problem is to improve the resin fluidity, which can be achieved using two methods. The first method is the use of solvents. However, it is impossible to prepare dense materials with low porosity without efficient removal of the solvent during the molding process. The second method is improvement of the resin fluidity by blending. Polymer blending is a good way to tailor the properties of blended systems that generally have a useful combination of properties derived from each component polymer. Various thermoplastic polymer blends and thermoset polymer blends have been developed and successfully applied. Raj [33] prepared phenolic-urea-epoxy blends that exhibit better mechanical properties and higher thermal stability compared to epoxy resin. Nishimura [34] studied the molecular structure of cyanate ester (NCE)–epoxy blends and discussed the irradiation effect of gamma rays and fast neutrons; they found that the blended resin could survive a design period in a radiation environment. Harvey [35] prepared a homogenous polycarbonate/cyanate ester network and suggested that this kind of blend may have utility in the fabrication of toughed composite structures. Augustine [18] prepared hydroxyl-terminated, polyetheretherketone (PEEK)-toughed epoxy-amino novolac phthalonitrile blends; PEEK reduced the brittleness and improved their shear strength. Dominguez [36] separately blended epoxy resin with biphenyl PN and *n* = 4 PN and studied the cure behavior of the blends. The results showed that phthalonitrile–epoxy blends exhibited good processability and the copolymers had enhanced high-temperature properties (the *T_g_* temperature was 230 °C) compared with cured epoxy resin.

However, while the process of blending phthalonitrile monomers with epoxy resin has been improved, the thermal resistance of the resulting blend is much lower than that of phthalonitrile. Here, we blended the phthalonitrile–polyhedral oligomeric silsesquioxane (POSS)–modified phthalonitrile copolymer with novolac cyanate ester resin (NCE), which has higher thermal resistance. POSS-modified phthalonitrile and highly thermal-resistant NCE resin have similar structures and hence are mutually soluble at high temperatures. Moreover, the active groups of POSS can chemically react with both types of resins to improve their compatibility. The resulting blend has improved processability and resulted in a higher resistance than epoxy resin blends. Therefore, it is necessary to study the blends of phthalonitrile and NCE, which have great potential for engineering applications.

However, research on blends of phthalonitrile and NCE is rare. Therefore, the goal of this study is to further investigate such blends, including their curing behavior, processability, compatibility, and thermal properties by using differential scanning calorimetry (DSC), Fourier-transform infrared (FTIR) spectroscopy, thermogravimetric analysis (TGA), and dynamic mechanical analysis (DMA). These data and rheological properties offer an experimental basis for future applications in carbon-fiber-reinforced composites.

## 2. Experimental Details

### 2.1. Materials

**Figure 1:**
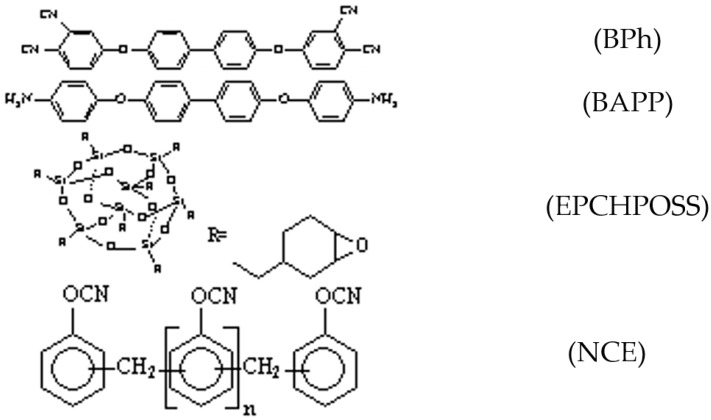
Structures of the phthalonitrile monomer, curing agent, phthalonitrile–polyhedral oligomeric silsesquioxane (POSS), and cyanate ester (NCE) used in this work.

BPh (solid, 99%, without any further purification) was synthesized at the Institute of Chemistry of the Chinese Academy of Sciences, Beijing, China. 4,4′-bis(4-aminophenoxy)biphenyl (BAPP, solid, without any further purification) was purchased from Bailingwei, Inc. (J&K Scientific Ltd., Beijing, China). The epoxycyclohexyl polyhedral oligomeric silsesquioxane (EPCHPOSS, semi-solid, without any further purification) was obtained from Hybrid Plastics, Inc. (Hybrid Plastics, Fountain Valley, CA, USA). NCE resin (liquid, without any further purification) was purchased from Lonza (China) Investments Co., Ltd. (Lonza Ltd., Visp, Switzerland). The structures of the phthalonitrile monomer, curing agent, POSS, and NCE are shown in Figure 1.

### 2.2. Preparation of Cyanate and POSS-Phthalonitrile Blends

BPh was melted at 260 °C and BAPP (curing agent, 2 wt.%) was added with continuous stirring. EP0408 was then added at 0.5 wt.% (the concentration shown to have the best thermal stability in our previous work [27]) to the mixture with stirring and then cooled to room temperature; this was named the EPCHPOSS–BPh prepolymer. The EPCHPOSS–BPh prepolymer was pulverized into powder and added to NCE resin at varying compositions (10, 20, 30, and 40 wt.%); the mixtures were named 1090, 2080, 3070, and 4060 prepolymers, respectively. All prepolymers were cured at 280 °C (4 h), 300 °C (8 h), and 325 °C (8 h); these were then subsequently post-cured under an inert N_2_ atmosphere at 350 °C (4 h) and 375 °C (2 h). The cured samples were called EPCHPOSS–BPh polymer and 1090, 2080, 3070, and 4060 polymers. The prepolymers were pulverized before performing DSC and rheological tests and studied by TGA, FTIR, and DMA.

### 2.3. Characterization

DSC experiments were conducted in a flowing N_2_ atmosphere on EPCHPOSS–BPh prepolymer and 1090, 2080, 3070, and 4060 prepolymers. The experiments were conducted in a Perkin–Elmer Pyris-6 DSC calorimeter (Perkin–Elmer, Richmond, CA, USA) at a heating rate of 10 °C/min. The DSC curves at different heating rates (10, 20, 30, and 40 °C/min) were measured. The activation energies were calculated using the following equation:

(1) $$\frac{d\left[\mathrm{In}\left(\beta /T_{p}^{2}\right)\right]}{d\left[1/T_{p}\right]}=-\frac{E}{R}$$

where *β* is the heating rate, *T_p_* is the peak temperature of each DSC curve at different heating rates, and *R* is the universal gas constant. Thus, the *E* value was obtained through the linear dependence of *In*(*β*/*T_p_*^2^) on 1/*T_p_* at various heating rates.

EPCHPOSS–BPh polymer as well as 1090, 2080, 3070, and 4060 polymers and NCE polymer were pulverized into powder and mixed with spectroscopy grade KBr to make pellets for FTIR. Their chemical structures were studied using a FT-IR spectrometer(Nicolet Avatar 370, ThermoFisher Scientific, Grand Island, NY, USA) with potassium bromide pellets containing a low concentration of sample; the wavenumber range was 500–4000 cm^−1^ at a resolution of 4 cm^−1^. Thermal analysis was conducted on the polymers using a thermogravimetric analyzer (SDTQ600, TA Instruments, Eden Prairie, MN, USA). TGA tests were performed in air at a scan rate of 10 °C/min and a flow rate of 100 mL/min. The dynamic storage modulus (*G*′) and damping factor (tanδ) of the rectangular phthalonitrile polymer specimens (50 × 10 × 3 mm) were obtained using a DMA instrument (DMS-6100, NSK Ltd., Tokyo, Japan) with a N_2_ atmosphere and a temperature of 30–400 °C (rate 4 °C/min, frequency 10 Hz). Thus, the *T_g_* temperature was estimated from the modulus–temperature plots obtained by DMA. Dynamic viscosity measurements were performed on a rheometer (AR-2000, TA Instruments, Eden Prairie, MN, USA). The samples were placed on the platforms and heated from 80 to 280 °C under the fixed strain values of 50% with a fixed frequency of 1 rad/s. The dynamic viscosity and shear storage modulus were obtained.

## 3. Results and Discussion

**Figure 2:**
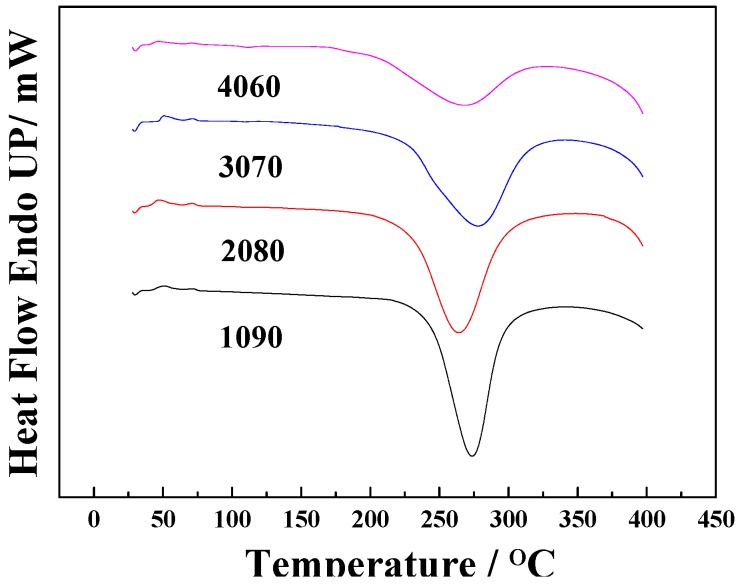
Differential scanning calorimetry (DSC) scanning diagram of 1090, 2080, 3070, and 4060 blend resins.

The 1090, 2080, 3070, and 4060 resin blends were subjected to DSC scanning from room temperature to 400 °C (Figure 2).

The DSC curves of the 1090, 2080, 3070, and 4060 resin blends each have a large exothermic reaction peak. This is very different from the DSC curve of the previously studied POSS-modified phthalonitrile [22]. This difference is attributed to the much higher exotherm of NCE [37,38] during the reaction than that of phthalonitrile resin. This masks the endothermic and exothermic peaks in the DSC curves of phthalonitrile resins. As a result, the phthalonitrile content increased. The intensities of the exothermic peaks associated with the curing reaction of NCE became smaller, and the corresponding enthalpy values were −569, −507, −465, −367, and −303 J/g, respectively. The decrease in heat release alleviated the implosion of cyanate resin due to the large amount of released heat. The initial reaction temperatures of the 1090, 2080, 3070, and 4060 resin blends gradually decreased as the phthalonitrile content increased, i.e., 243, 228, 225, 201, and 180 °C, respectively. This indicates that the initial reaction proceeded more easily as the phthalonitrile content increased, but this was accompanied by a decreased reaction intensity, as reflected by the decrease in exothermic energy.

The Kissinger equation [39] was used to calculate the activation energies of the 1090, 2080, 3070, and 4060 resins as 82, 75, 140, and 108 kJ/mol, respectively. Except for the 3070 resin, the other three resins had significantly lower activation energies than phthalonitrile. Activation energy indicates the reactivity of a system. Lower activation energy leads to a higher reactivity, showing that the introduction of the NCE resin significantly improved the reactivity. However, the reactivity began to decrease significantly upon addition of more than 30 wt.% of phthalonitrile. This finding shows that the addition of a small amount of phthalonitrile promoted polymerization reactions; when the contents of the two resins were comparable, their respective polymerization reactions affected each other.

**Figure 3:**
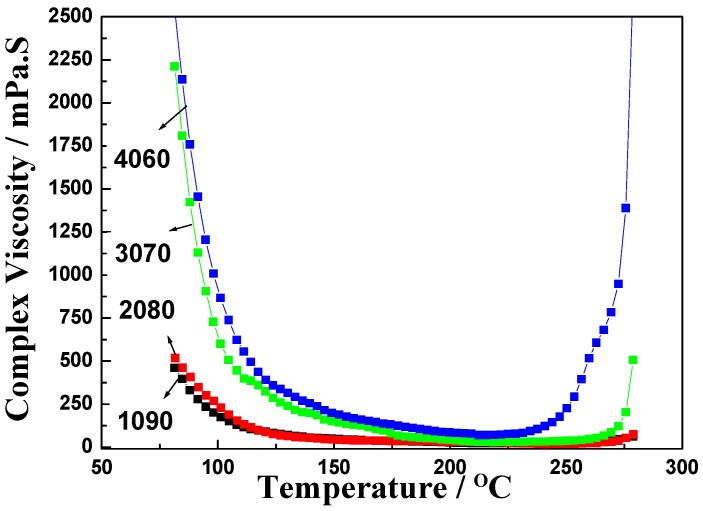
Complex viscosity of blends as a function of temperature. (**a**) 1090, (**b**) 2080, (**c**) 3070, and (**d**) 4060.

The rheological properties of the 1090, 2080, 3070, and 4060 resin blends were investigated [40,41], and the results are shown in Figure 3. As the temperature increased, their viscosities all first decreased and then reached a steady state before rapidly increasing. The lowest viscosities of the 1090, 2080, 3070, and 4060 resin blends were 17, 25, 32, and 89 mPa·S, respectively. The viscosities of the 1090, 2080, and 3070 resin blends were very low. However, after the content of EPCHPOSS–BPh prepolymer increased to 40%, the viscosities of the blends increased rapidly to 89 mPa·s, respectively. The temperature at which the viscosity started to increase again from a steady state is termed the gel point of the system; that is, the temperature at which the polymer starts to cure. The gel-point temperatures of the 1090, 2080, 3070, and 4060 resin blends were 278, 278, 271, and 243, respectively, demonstrating that the gel-point temperature decreased as the content of the EPCHPOSS–BPh prepolymer increased. By adjusting its viscosity via temperature control, one can make a blend suitable for the molding of carbon-fiber-reinforced composites in the winding and prepreg lay-up processes. Compared to phthalonitrile, the processability of the blends was greatly improved, leading to more extensive applications of the resin matrix.

**Figure 4:**
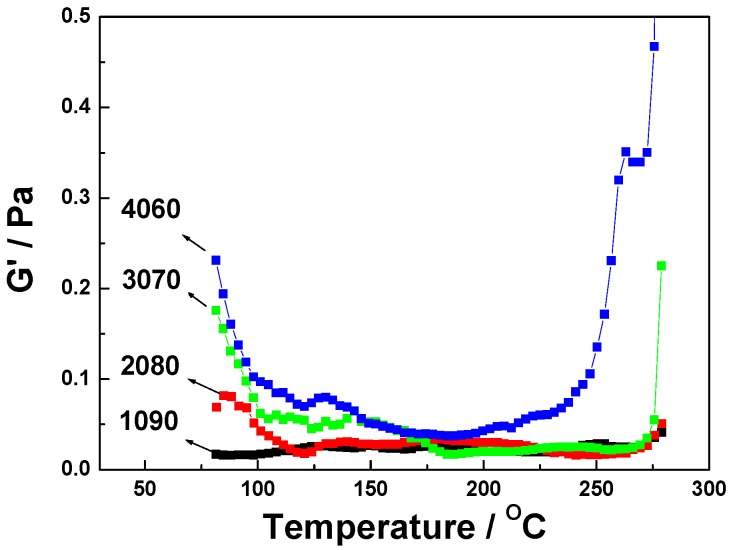
Shear storage modulus (G’) as a function of temperature curves for 1090, 2080, 3070, and 4060 resins.

The shear storage modulus as a function of temperature curves for 1090, 2080, 3070, and 4060 resins are shown as Figure 4. As the temperature increases, the storage modulus first decreased, then stabilized, and then finally increased quickly. The trend of storage modulus change is consistent with the trend of viscosity change. A lower viscosity leads to greater fluidity. This makes chain movement easier, leading to a smaller modulus. The modulus increased with increasing content of EPCH–BPh prepolymer; the modulus began to increase quickly when the temperature approached the gel temperature.

**Figure 5:**
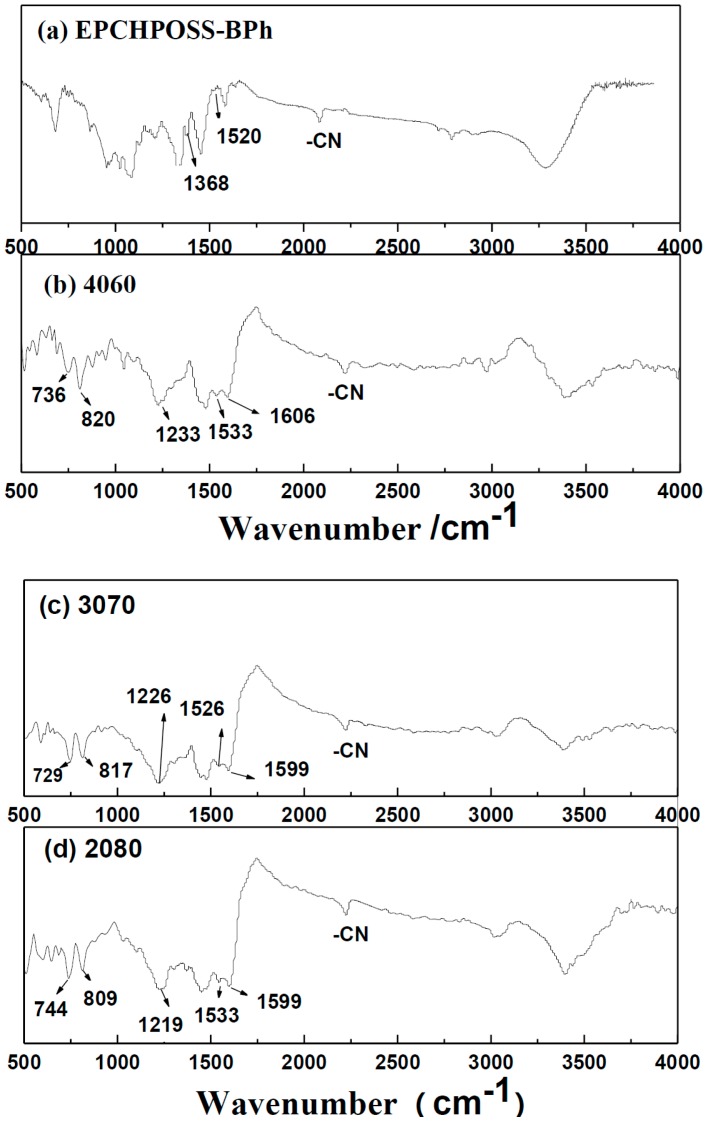

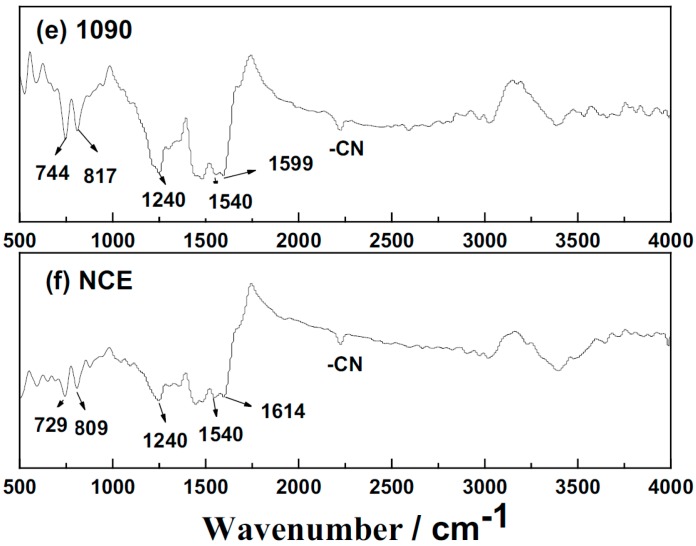
Fourier-transform far-infrared (FTIR) spectra of NCE, EPCHPOSS–BPh, 1090, 2080, 3070, and 4060 copolymers.

The structures of the NCE resin polymer, the 1090, 2080, 3070, and 4060 resin blend polymers, and the EPCHPOSS–BPh polymer were studied and compared in Figure 5. The FTIR spectrum of the copolymer is more similar to that of NCE.

In the FTIR spectra of the NCE, 1090, 2080, 3070, and 4060 polymers, the 729-, 744-, 744-, 729-, and 736-cm^−1^ peaks are δCH (out-of-plane deformation) of 1,2,3-trisubstituted benzene; the 809-, 817-, 809-, 817-, and 820-cm^−1^ peaks are δCH (out-of-plane deformation) of the triazine compound; the 1240-, 1240-, 1219-, 1226-, and 1233-cm^−1^ peaks are γC-OC of the ether bond; the 1540-, 1540-, 1533-, 1526-, and 1533-cm^−1^ peaks are γ rings of the triazine ring; and the 1614-, 1599-, 1599-, 1599-, and 1606-cm^−1^ peaks are benzene rings of the aromatic ring [42,43].

**Figure 6:**
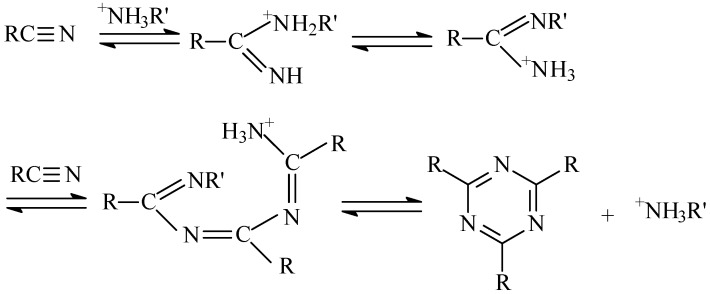
The mechanism of generating a triazine ring [44].

**Figure 7:**
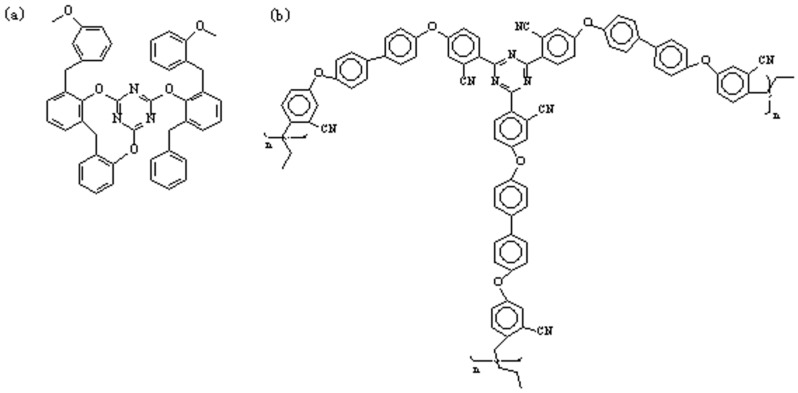
The possible structure of the blends: (**a**) Structure of cured NCE; (**b**) Structure of cured phthalonitrile.

The structure of the EPCHPOSS–BPh polymer is mainly composed of triazine rings in which the C atoms are linked by aromatic compounds. In comparison, the structure of the NCE resin is different in that its triazine rings are linked by ether bonds. According to Burchill [44], polymeric products of —CN include the polymer with a triazine ring as the crosslinking point when organic amines and ammonium organic acid salts are used. The mechanism is shown in Figure 6. The structures of the 1090, 2080, 3070, and 4060 copolymers are interpenetrating polymer networks with two types of triazine rings (Figure 7). The structure of the NCE polymer shows that the links of the triazine ring to the ether bond increase the flexibility of the molecular chain, thus lowering the thermal resistance versus the EPCHPOSS–BPh polymer. The introduction of POSS tightly links the network structures of the two polymers.

**Figure 8:**
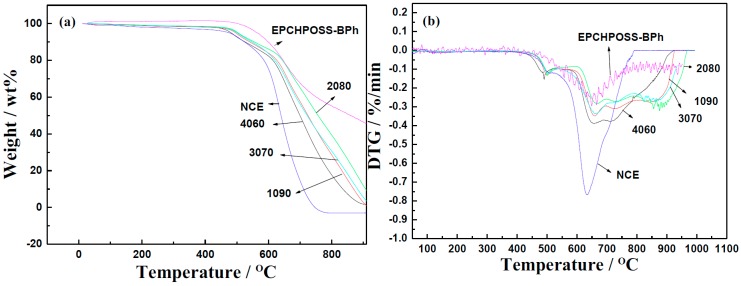
Thermogravimetric analysis (TGA) and Derivative (DTG) plots of NCE, EPCHPOSS–BPh, 1090, 2080, 3070, and 4060 polymers in the air. (**a**) TG curves and (**b**) DTG curves.

The TG properties of NCE resin polymer, 1090, 2080, 3070, and 4060 resin polymer blends, and EPCHPOSS-BPh polymer were studied and compared [10,45]. The changes in their Thermogravimetry (TG) curves and Derivative Thermogravimetry (DTG) curves as a function of temperature are shown in Figure 8.

The thermal resistance of the NCE polymer is significantly lower than that of other polymers. The rapid decline in mass starting at 593 °C represents a significant difference between the NCE polymer and other polymers. The drastic weight loss causes the TG curve of the NCE polymer to be significantly lower than the other polymers. The temperatures at which the NCE polymer, 1090, 2080, 3070, and 4060 polymer blends, and EPCHPOSS–BPh polymer began to lose weight were 452, 476, 480, 477, 455, and 505 °C, respectively. The temperature at which the NCE polymer, 1090, 2080, 3070, and 4060 polymer blends, and EPCHPOSS–BPh polymer experienced 5% weight loss were 475, 496, 503, 500, and 564 °C, respectively. The resin blend with 20% EPCHPOSS–BPh polymer had the highest thermal resistance and showed a retention of 12% at 900 °C; the NCE polymer had 100% weight loss at this temperature. In addition, the EPCHPOSS–BPh polymer had the highest thermal resistance and showed a retention of 48% at 900 °C (shown in Table 1).

The DTG curves showed that the NCE polymer had the lowest thermal resistance and the largest peak (hence the highest rate) of weight loss. The EPCHPOSS–BPh polymer had the lowest rate of weight loss, followed by the 2080 polymer. There were two stages of the weight-loss rate in the DTG curves. A small weight-loss-rate peak appeared when the decomposition temperature was reached. The temperatures corresponding to the highest weight-loss rates of NCE polymer, 1090, 2080, 3070, and 4060 polymer blends, and EPCHPOSS–BPh polymer were 630, 660, 669, 662, 648, and 664 °C, respectively. As the temperature increased from that corresponding to the highest rate of weight loss, the weight-loss rate of the 2080 polymer gradually decreased; those at 1090, 3070, and 4060 blends showed a plateau, indicating that these blends still continued to lose weight at their highest rates.

**Figure 9:**
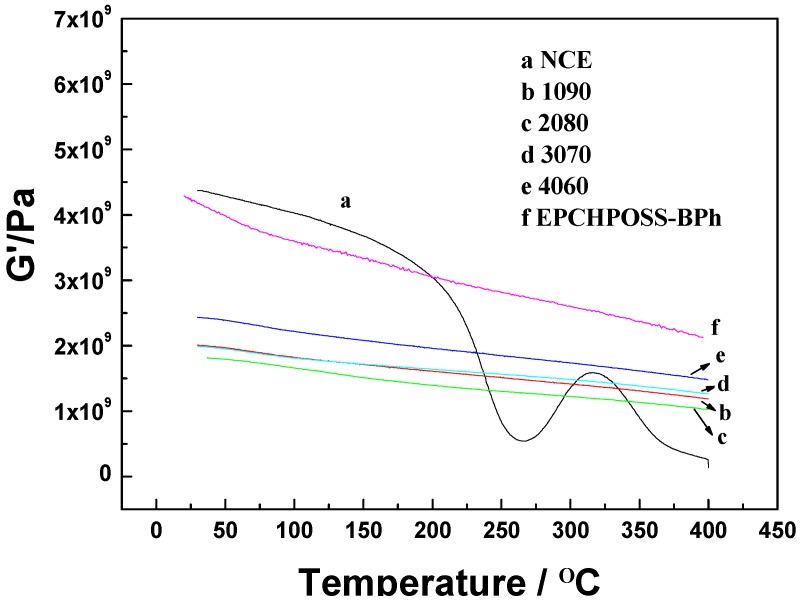
Dynamic storage modulus (G′) as a function of temperature for NCE, EPCHPOSS–BPh, 1090, 2080, 3070, and 4060 polymers under a nitrogenous atmosphere.

**Figure 10:**
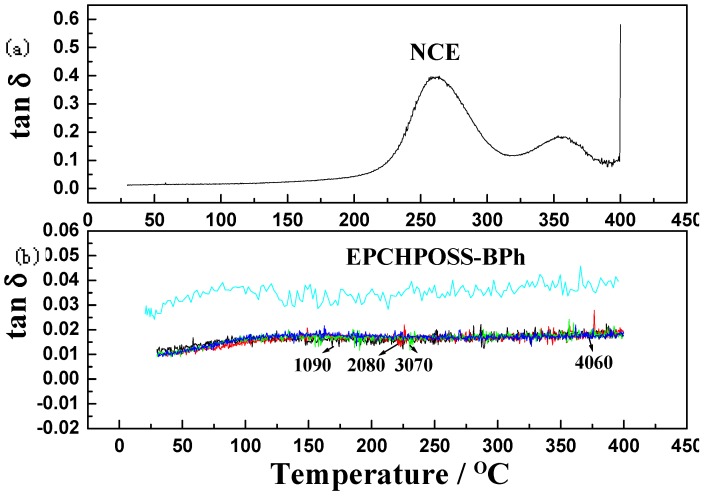
Damping factor (tanδ) as a function of temperature for NCE, EPCHPOSS–BPh, 1090, 2080, 3070, 4060 polymers in the nitrogenous atmosphere: (**a**) NCE, (**b**) EPCHPOSS–BPh, 1090, 2080, 3070, and 4060.

The NCE polymer, 1090, 2080, 3070, and 4060 polymer blends, and EPCHPOSS–BPh polymer were tested using DMA. The variations of dynamic storage moduli and damping factors (tanδ) with temperature are shown in Figure 9 and Figure 10, respectively.

Figure 9 shows that the dynamic storage moduli of the EPCHPOSS–BPh polymer and the 1090, 2080, 3070, and 4060 copolymers gradually decreased with increasing temperatures. This decrease was attributed to the stress relaxation of the polymer network structures. The moduli at room temperature were 4.4, 2.02, 1.89, 1.99, and 2.4 GPa, respectively. When the temperature increased to 400 °C, the moduli decreased to 2.4, 1.19, 1, 1.28, and 1.44 GPa, respectively, amounting to 54%, 58%, 53%, 64%, and 60% of the respective moduli at room temperature. Thus, the 3070 copolymer had the highest modulus retention rate.

The dynamic storage modulus of NCE varied with temperature in a significantly different manner. As the temperature increased, its modulus decreased rapidly from 4.5 GPa at room temperature to the lowest value of 0.52 GPa at 266 °C. From here, the temperature further increased, and its modulus increased moderately. As the temperature increased beyond 316 °C, its modulus decreased rapidly again and reached 0.2 GPa at 400 °C.

Figure 10 shows that the damping factors (tan δ) of EPCHPOSS–BPh polymer and 1090, 2080, 3070, and 4060 copolymers vary smoothly with increasing temperature without the presence of glass-transition peaks, indicating that all three types of polymers formed a stable three-dimensional network structure that prevented the slippage of chain segments as temperature increased. The absence of glass-transition peaks means that the glass-transition temperature was higher than 400 °C. There are two peaks at 259 and 356 °C. In the curve depicting the variation of the damping factor (tanδ) of NCE resin with temperature, these two peaks correspond to the two fluctuations in the variations of dynamic storage modulus with time in Figure 6. The two peaks are associated with the two phases in the curing process, indicating that the polymer chain of the NCE resin underwent a glass transition starting at 259 °C, resulting in the slippage of chain segments. This slipped further at 356 °C. A thorough comparison shows that the thermal resistance of NCE blends significantly improved, and the glass-transition temperature of the blends was at least 140 °C higher than that of NCE [10,11,36].

**Figure 11:**
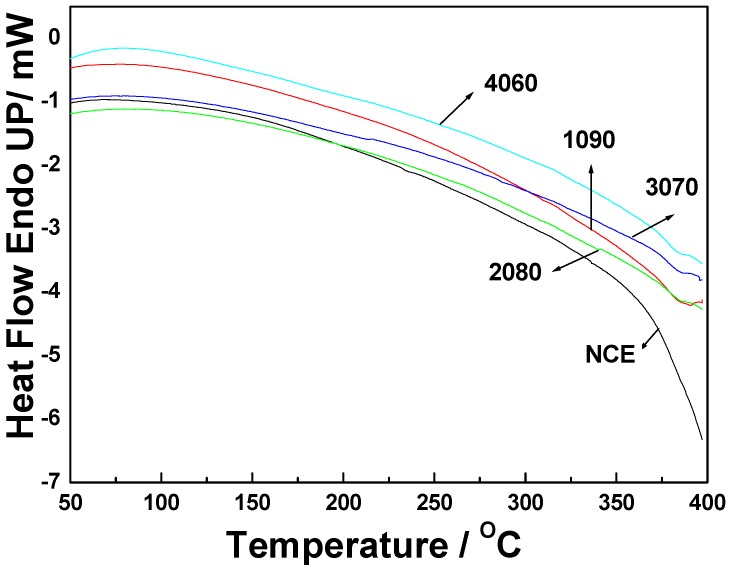
DSC scanning of NCE, 1090, 2080, 3070, and 4060 polymers under nitrogenous atmosphere.

The *T*_g_ temperatures were obtained from DMA for NCE, 1090, 2080, 3070, and 4060 polymers. This was included with DSC data in Figure 11. All curves undergo exothermic processes when the temperature is elevated—especially the NCE polymer, which had the highest heat release. Figure 11 shows that 2080 had no obvious *T*_g_ temperature (above 400 °C), while the *T*_g_ temperatures of NCE, 1090, 3070, and 4060 were 360 °C, 384 °C, 380 °C, and 377 °C. These were different from the DMA results. The cure extent of the polymer and the aged extent [46] at elevated temperatures will affect the *T*_g_ temperature obtained from DSC.

## 4. Conclusions

The following conclusions can be drawn from the above analysis:

Conclusion 1: The DSC results indicate that as the content of phthalonitrile increased, the exothermic energy of the blend gradually decreased. This alleviates the implosion due to the large amount of heat released from cyanate resin. The activation energies of the 1090, 2080, 3070, and 4060 blends were 81.6, 75.2, 140.3, and 108 kJ/mol, respectively. Except for the 3070 resin, the other three resins had activation energies that were significantly lower than that of phthalonitrile, indicating that the introduction of NCE resin significantly improves the reactivity.

Conclusion 2: The FTIR results indicate that the structures of the 1090, 2080, 3070, and 4060 copolymers were interpenetrating polymer networks with two types of triazine rings. The introduction of POSS tightly linked the network structures of the two types of polymers.

Conclusion 3: The TGA results show that the resin blend with 20% of EPCHPOSS–BPh prepolymer had the highest thermal resistance and showed a retention of 12% at 900 °C.

Conclusion 4: DMA results show that the thermal resistance of the NCE blends were significantly improved, and the glass-transition temperature of the blend was at least 140 °C higher than that of NCE resin.

Conclusion 5: Investigation of the rheological properties shows that the viscosity of the resin blend was still very low and can be adjusted by controlling the temperature. This makes the resin blend suitable for molding the carbon-fiber-reinforced composites in the winding and prepreg lay-up processes. Compared to phthalonitrile, the processability of the resin blend was greatly improved. This suggests extensive applications for this resin matrix.

## Figures and Tables

**Table 1:** Thermal stability of EPCHPOSS–BPh, 1090, 2080, 3070, 4060, and NCE polymers.

Properties	EPCHPOSS–BPh	1090	2080	3070	4060	NCE
T_5%_ (°C)	564	496	503	500	481	475
Char yield (%)	48	2.7	12	5.5	1.8	0
T_lost fast_ (°C)	664	660	669	662	648	630

T_5%_: temperature of 5% weight loss in a normal air atmosphere. Char yield: percentage of polymer. T_lost fast_: temperature of loss fast.
